# Attenuation of eccentric exercise-induced muscle damage conferred by maximal isometric contractions: a mini review

**DOI:** 10.3389/fphys.2015.00300

**Published:** 2015-10-27

**Authors:** Leonardo C. R. Lima, Benedito S. Denadai

**Affiliations:** Human Performance Laboratory, Department of Physical Education, Biosciences Institute, São Paulo State UniversityRio Claro, Brazil

**Keywords:** exercise-induced muscle damage, isometric contractions, prevention strategy, repeated bout effect, pre-conditioning

## Abstract

Although, beneficial in determined contexts, eccentric exercise-induced muscle damage (EIMD) might be unwanted during training regimens, competitions and daily activities. There are a vast number of studies investigating strategies to attenuate EIMD response after damaging exercise bouts. Many of them consist of performing exercises that induce EIMD, consuming supplements or using equipment that are not accessible for most people. It appears that performing maximal isometric contractions (ISOs) 2–4 days prior to damaging bouts promotes significant attenuation of EIMD symptoms that are not related to muscle function. It has been shown that the volume of ISOs, muscle length in which they are performed, and interval between them and the damaging bout influence the magnitude of this protection. In addition, it appears that this protection is not long-lived, lasting no longer than 4 days. Although no particular mechanisms for these adaptations were identified, professionals should consider applying this non-damaging stimulus before submitting their patients to unaccustomed exercised. However, it seems not to be the best option for athletes or relatively trained individuals. Future, studies should focus on establishing if ISOs protect other populations (i.e., trained individuals) or muscle groups (i.e., knee extensors) against EIMD, as well as investigate different mechanisms for ISO-induced protection.

## Introduction

Eccentric exercise-induced muscle damage (EIMD) is a multifactorial phenomenon that occurs when skeletal muscle is exposed to elevated mechanical stress conferred by unaccustomed eccentric exercise (Clarkson and Hubal, 2002). This exposure to stress leads to cellular disruption, loss of function, soreness, and leakage of intracellular proteins to the blood stream (Brentano and Martins Kruel, 2011).

Although EIMD plays an important role in neuromuscular development (Schoenfeld, 2012), some of its symptoms may acutely affect performance and the well-being of different populations. Recent studies showed that EIMD compromises efficiency (Assumpção et al., 2013), explosive force (Peñailillo et al., 2015), range of motion (Chen et al., 2011), and leads to soreness (Nelson, 2013).

Many interventions have been proposed to avoid the detrimental effects of EIMD. The most effective strategy identified so far is performing an initial damaging bout, after which the neuromuscular system recovers, and becomes less susceptible to EIMD. This phenomenon is known as the repeated bout effect (RBE) (McHugh, 2003). However, the manifestation of elevated magnitudes of EIMD seems not to be necessary for this protection to occur.

One protective strategy that has gained scientific attention is performing maximal isometric contractions (ISOs) few days before damaging bouts (Chen et al., 2013). There is evidence that this type of intervention might be a viable non-damaging strategy against EIMD. Considering this, the aim of the present study was to critically review the literature on the use of ISOs as a prevention strategy against EIMD.

## Exercise-induced muscle damage and the repeated bout effect

The breakdown of ultra-structural components of the muscle induced by unaccustomed eccentric exercises is referred as EIMD. This phenomenon has been widely studied in an effort to understand its beneficial (Schoenfeld, 2012) and detrimental (Howatson and van Someren, 2008) effects on performance and health. The most commonly reported EIMD symptoms are losses in muscular function, edema, soreness, stiffness, and leakage of intracellular proteins to the blood stream (Clarkson and Hubal, 2002).

When compared to isometric and concentric, eccentric contractions are known to cause significantly greater magnitudes of damage (Nosaka and Newton, 2002). This difference in the response within different types of contraction seems to be related to different recruitment patterns. Although eccentric contractions produce greater strength levels when compared to other contractions, fewer motor units are recruited during them (Enoka, 1996), resulting in greater mechanical stress applied to active muscle fibers and non-contractile structures. Thus, when elevated tension is applied to contractile and non-contractile structures, disruption and disorganization occur, leading to compromised contractile function (Brentano and Martins Kruel, 2011).

Elevated levels of mechanical stress imposed upon muscle fibers during eccentric contractions can also compromise organelles and cell membrane, leading to disturbances in myocite homeostasis (Clarkson and Hubal, 2002). As an example, excitation-contraction coupling is compromised after eccentric actions, leading to attenuated Ca^2+^ release by the sarcoplasmic reticulum and, consequently, strength loss (Corona et al., 2009). Moreover, mechanical damage to the sarcoplasmic reticulum can also increase Ca^2+^ concentration in the cytoplasm, which is known to activate proteases that promote further damage to contractile and non-contractile proteins (Verburg et al., 2005). This initial mechanical damage also stimulates the release of pro-inflammatory cytokines that signals for a secondary damage event (for review, see Paulsen et al., 2012).

The secondary damaging event occurs as a response of the immune system to initial damage. Leukocytes migrate to the damaged site and promote damage through phagocytosis before signaling for tissue reconstruction (Hirose et al., 2004). This inflammatory response is responsible for the manifestation of symptoms like swelling and soreness (Howatson et al., 2010). It also plays a minor role in strength loss, since reactive oxygen species secreted by neutrophils and macrophages are believed to promote peroxidation of the membrane and contractile proteins of nearby healthy myocites (Hirose et al., 2004).

It has been reported that a second damaging bout does not elicit EIMD symptoms to the same extent after recovery from a first, identical, bout. This attenuated response has been attributed to a phenomenon often referred as the RBE. Although, many studies focused on discovering the mechanisms responsible for the RBE, they are yet to be fully determined.

In an effort to understand the RBE, McHugh (2003) reviewed the literature on the possible mechanisms of this phenomenon and divided them into three groups: neural, mechanic, and cellular adaptations. The studies that proposed neural adaptations showed that greater numbers of slow twitch fibers are recruited during eccentric contractions following an initial damaging bout. Moreover, motor units are recruited in a more synchronized fashion (McHugh et al., 2001). As for mechanic adaptations, augmented muscular stiffness has been reported after recovery from initial damage (Lacourpaille et al., 2014), as well as increases in connective tissue (Lapier et al., 1995) and total desmin (a structural protein responsible for myofibril alignment) content (Barash et al., 2002). Finally, studies that investigated cellular adaptations found increased numbers of serial sarcomeres in myofibrils (Brocket et al., 2001), strengthening of the sarcoplasmic recticulum (Clarkson and Tremblay, 1988) and altered expression of mediators of inflammation (Hubal et al., 2008).

Although, important in determined situations, the protection provided by the RBE might be contraindicated in given contexts, since it requires initial damage to occur. In some competitive contexts, severe EIMD is usually avoided, since it can compromise training sessions and proper training intervals. EIMD can also compromise adherence to physical activity programs because of soreness (Gabbe et al., 2006). In other words, submitting an individual to elevated levels of EIMD in order to protect him/her against it later might not always be the best option. Therefore, protective strategies that induce no EIMD should be considered.

## Maximal isometric contractions: a prevention strategy

Amidst many other strategies used to attenuate EIMD, performing ISOs few days before damaging bouts seems to be an efficient non-damaging alternative. Along with preconditioning concentric (Nosaka and Clarkson, 1997) and submaximal eccentric (SubECC—Chen et al., 2012a) contractions, this strategy gained scientific attention recently in a quest for strategies that would protect against EIMD eliciting low-to-none damage to the muscle. ISOs might be an efficient non-damaging alternative to promote acute protection against EIMD. A detailed review of the existing literature on this protection strategy, highlighting methodological aspects and outcomes on EIMD is presented on Table 1 and briefly explained in the next paragraphs.

**Table 1:** **Summary of studies that investigated the effects of performing maximal isometric (ISO), submaximal (SubECC), and maximal eccentric (MaxECC) contractions before bouts of eccentric exercise on the most commonly assessed markers of exercise-induced muscle damage**.

**Study**	**Preconditioning**				**Interval**	**Damaging bout**			
	**Preconditioning Protocol**	**Outcome**				**Damaging Protocol**	**Outcome**		
		**Strength (%)**	**CK (U/l)**	**Soreness (mm)**			**Strength (%)**	**CK (U/l)**	**Soreness (mm)**
Chen et al., 2013	CON	–	–	–	–	30 MaxECC at 90°.s^−1^	60	1924	57
	2 ISO	99^*^	118^*^	0^*^	2–4 days		63–66 (5–10)	478–976 (49–75)	32–42 (26–43)
Chen et al., 2012b	CON	–	–	–	–	30 MaxECC at 90°.s^−1^	58	1872	57
	2 and 10 ISO	99–100	116–117	0–1	2 days		62–69 (7–19)	236–445 (76–87)	21–35 (38–63)
Chen et al., 2012c	CON	–	–	–	–	30 ECC at 100%1RM	58	1742	46
	30 ISO at 20° and 90°	81–93	187–367	8–15	3 weeks		58–67 (0–15)	832–1716 (1–52)	16–35 (24–64)
Koh and Brooks, 2001	CON	–	N/A	N/A	–	75 MaxECC	45	N/A	N/A
	75 ISO	95	N/A	N/A	2 weeks		65 (44)	N/A	N/A
Chen et al., 2012a	CON	–	–	–	–	30 MaxECC at 30°.s^−1^	64	1917	53
	30 SubECC at 10%1RM	97	124	3	2 days		74 (16)	925 (52)	13 (75)
Chen et al., 2012c	CON	–	–	–	–	30 ECC at 100%1RM	58	1742	46
	30 SubECC at 10–20%1RM	80–88	350–490	11–20	3 weeks		59–64 (2–10)	1040–1378 (21–40)	17–21 (54–63)
	30 ECC at 100%1RM	60	1908	39	3 weeks		78.75 (36)	156 (91)	11 (76)
Nosaka et al., 2005	CON	-	-	-	-	24 MaxECC	43	6694	33
	24 MaxECC	54	6694	33	4 weeks		67 (56)	315 (95)	13 (61)

*Outcomes are expressed as mean values obtained immediately and 1–3 days after the damaging and preconditioning bouts. In parenthesis, percentages of protection are expressed compared to the control group. To calculate the percentage of protection, absolute values for the control groups were subtracted from those of the experimental groups, normalized by the control group value and multiplied by 100*.

* *mean of the 2- and 4-day groups values. 1RM, one repetition maximum; ECC, eccentric contraction; CK, creatine kinase activity; N/A, not assessed*.

To our knowledge, the first study to investigate the effects of pre-conditioning ISOs on EIMD was conducted by Koh and Brooks (2001), who found significant protection against EIMD in mice conferred by performing 75 ISOs 2 weeks before a damaging bout. Strength loss was significantly lower (2/3 of CON) in the group that performed the ISOs with no significant difference in histological damage. This study was the first to evidence that protection against EIMD can be conferred without manifestation of previous damage. It also showed that the loss of function elicited by EIMD might not be uniquely related to histological damage.

Few studies investigated the protective effects of ISOs against EIMD in humans and, to our knowledge, all have been published by the same research group (Chen et al., 2012b,c, 2013). The authors manipulated important aspects that have been shown to interfere in the magnitude of EIMD and the resulting protection conferred by it in previous maximal eccentric contraction (MaxECC) models (Nosaka et al., 2001a, 2005; Lima and Denadai, 2011). For instance, Chen et al. (2013) found that the protection conferred by 2 ISOs is not longed lived, lasting up to 4 days with a peak of protection occurring 2 days after the preconditioning. They also found that it takes at least 2 days for this protective effect to manifest. However, when 30 ISOs were performed, Chen et al. (2012c) found that the resulting protection lasts up to 3 weeks. Therefore, it seems that the lasting of ISO-induced protection is volume-dependent.

Furthermore, Chen et al. (2012b) showed that the magnitude of prophylaxis against EIMD induced by ISOs is also volume-dependent. In their study, although both ISO volumes conferred significant protection, subjects that performed 10 ISOs presented greater protection against EIMD than those who performed 2 ISOs. This volume-protection relationship seems not be linear, since the protection conferred by 10 ISOs was approximately 2 times greater than that conferred by 2 ISOs (a 5 times smaller volume). This same relationship seems to occur in MaxECC preconditioning (Nosaka et al., 2001a), with a volume limit (6 ECCs) after which protection seems not to be greater. Although, similar in the volume-protection relationship, ISO and MaxECC prophylaxis are substantially different in duration, with the second lasting noticeably longer than the first (Nosaka et al., 2001b). Finally, Chen et al. (2012c) showed that ISOs performed at long muscle lengths (20° elbow flexion) protect more than those performed at short muscle lengths (90°). The same muscle length-protection relationship has been identified for MaxECC prophylaxis (Nosaka et al., 2005).

**Figure 1:**
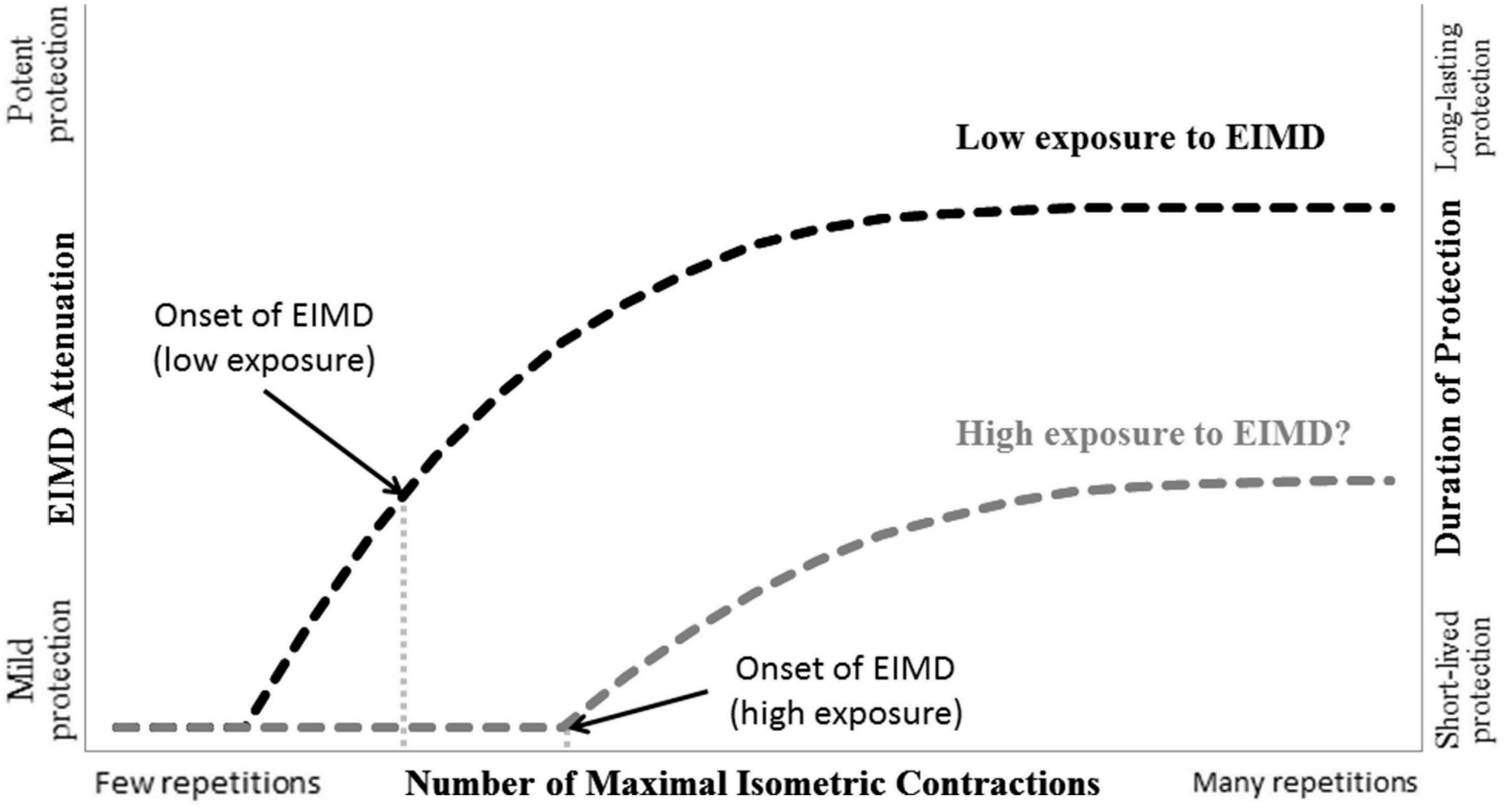
**Schematic illustration of the hypothetical relationship between the number of maximal isometric contractions (ISOs), attenuation of exercise-induced muscle damage (EIMD), and duration of the conferred protection according to the data from Chen et al. (2012b,c, 2013)**. Note that muscle groups that are not frequently exposed to EIMD (e.g., untrained and/or upper limb muscles) might benefit from the protective effect conferred by ISOs without the manifestation of EIMD (i.e., before the onset of EIMD). Based on data obtained from maximal eccentric contractions (Chen et al., 2011; Lima and Denadai, 2011), we also speculate that muscle groups that are highly exposed to EIMD (e.g., trained and/or lower limb muscles) require a greater number of ISOs in order to benefit from a protective effect with lesser magnitude and duration and that EIMD is required for it to manifest. The “?” represents that this assumption is hypothetical and based on studies that did not use ISOs as a preconditioning activity.

One of the advantages of adopting ISOs as a prophylactic strategy against EIMD is its non-damaging characteristic. Indeed, only one group of the above mentioned studies presented significant alterations in EIMD markers after ISOs (Chen et al., 2012c) due to the elevated volume of contractions (i.e., 30). Taken together, these data support our hypothetical model presented in Figure 1, which proposes a relationship between the number of ISOs and the resulting magnitude and lasting of protection.

To better understand the protective effects of ISO, it is necessary to compare them to other protective strategies that might (MaxECC) or might not (SubECC) induce significant EIMD. Table 1 shows the absolute values and percentage of protection conferred by ISOs (Koh and Brooks, 2001; Chen et al., 2012b,c, 2013), submaximal (Chen et al., 2012a,c), and maximal (Nosaka et al., 2005) eccentric contractions in the most commonly assessed EIMD markers (i.e., strength production, creatine kinase activity and soreness) over a 3-day period following the preconditioning activities and damaging bouts. Based on evidence from these studies, it seems reasonable to assume that the most potent ISO protocols might confer protection levels as great as those conferred by SubECC without eliciting significant EIMD. Indeed, Chen et al. (2012c) verified that the magnitude of the protection conferred by ISOs and SubECC is similar whereas the second elicits significant EIMD. However, both ISOs and SubECC present robust attenuations in soreness and creatine kinase activity (CK) responses, but mild protection against strength loss. When compared to MaxECC preconditioning (Nosaka et al., 2005), these prophylactic strategies present similar levels of protection in markers not related to muscle function with the protective effect conferred by MaxECC being noticeably greater for muscle function. The protection conferred by MaxECC also lasts longer (up to 9 months) (Nosaka et al., 2001b). This difference in protection magnitude and lasting seems to be in accordance to what was proposed by Fridén and Lieber (1992), that MaxECC affects the largest and most difficult to recruit fibers.

Finally, even though statistically significant protection was achieved for EIMD markers in all the studies that investigated ISOs as a protective strategy (Koh and Brooks, 2001; Chen et al., 2012b,c, 2013), one must question its usefulness in practical situations. It is somewhat intriguing that the protective effect conferred by ISOs seems to be more pronounced in markers (i.e., CK and soreness) that are not related to muscle function (i.e., strength loss). Moreover, Brancaccio et al. (2007) verified that increases in CK are not associated to loss of muscle function. This uneven response between EIMD markers should be considered by professionals in order to avoid wrongful associations of these markers in the context of preventing losses in muscle function, as an example.

## Possible physiological mechanisms

As the reviewed studies were pioneers in this field of investigation, no particular physiological mechanisms were assessed. However, hypotheses about potential mechanisms behind this protection were formulated based on other models that have been more deeply investigated. One particular mechanism that was proposed is strengthening of the extracellular matrix (Chen et al., 2012b). Mackey et al. (2011) showed that deadhesion of the extracellular matrix happens after a first damaging bout, leading to an adaptive response and consequent strengthening and attenuation of deadhesion after a second bout. However, in their study ISOs elicited significant EIMD. Whether extracellular matrix strengthening happens in the absence of histological damage is yet to be determined.

Another plausible mechanism for ISO-induced protection against EIMD is based on changes in the expression of genes related to the response to reactive oxygen species. McArdle et al. (2004) found that, after performing a bout of ISOs, gene expression of the haem-oxygenase-1 enzyme was increased, providing a protective effect against oxidative stress-induced damage in rats. Interestingly, Sloboda and Brooks (2013) found that reactive oxygen species production is similar between ISOs and eccentric contractions at similar effort levels, which means that performing ISOs (maximal) leads to a more oxidative status than performing SubECC, possibly leading to greater oxidative stress-related protection (Chen et al., 2012c). Similar protection against exercise-induced oxidative stress has already been reported elsewhere (El Abed et al., 2011). Up-regulation of heat shock proteins could also play an important role in protection against EIMD. In fact, McArdle et al. (2001) found increased expression of heat shock proteins after performing high volumes of ISOs.

Considering a possible inflammatory protection mechanism induced by ISOs, Pizza et al. (2001) found significant increases in neutrophil accumulation in rat muscles after ISOs without manifestation of histological damage. Two weeks later, the rats that performed ISOs presented blunted increases in neutrophil accumulation after eccentric contractions. In fact, using a different preconditioning strategy (passive stretching), Lockhart and Brooks (2008) showed that neutrophils contribute to adaptations that protect muscles from injury.

Another possible mechanism that could explain the protective effect conferred by ISOs is related to muscular stiffness (please see, Lacourpaille et al., 2014 for better comprehension on muscular stiffness). McHugh et al. (1999) identified a negative correlation between muscle compliance and susceptibility to muscle damage, by dividing subjects in groups according to their hamstrings muscles stiffness. Those who were stiffer presented significantly greater alterations in EIMD markers after a damaging bout than those who were more compliant. Additionally, Kay and Blazevich (2009) found acute decreases in muscle-tendon stiffness of the plantar flexors after a bout of ISOs, lasting up to 30 min, when their last measurement was performed. If this complacence-inducing effect were to last up to 2–4 days, it could act as another mechanism behind the ISOs-induced protection against EIMD.

The protection conferred by ISOs can also be a result of neural adaptations such as an optimization of the activation of agonist and antagonist motor units. Green et al. (2014) reported that performing 3 sets of 5 maximal isometric contractions resulted in decreased activation of antagonist muscles and, more importantly, a simultaneous increase in agonist force production and activation 3 days later. This activation of a greater number of motor units in the eccentrically exercised muscle can result in dividing the tension within more muscle fibers, which could attenuate EIMD.

Overall, protection conferred by ISOs seems to be related to the above mentioned mechanisms that do not require previous damage. In part, this could explain the differences in the magnitude and duration of the protection when compared to MaxECC. The occurrence of EIMD after MaxECC seems to lead to different adaptations (e.g., addition of new sarcomeres in series) that may promote stronger protection against changes in indirect EIMD markers and, specially, strength loss. Authors should feel encouraged to explore new mechanisms and boundaries on ISO-induced protection. For instance, it is important to investigate if this type of pre-conditioning activity is also efficient in trained populations or other muscle groups of untrained people, considering that muscle groups that frequently perform eccentric contractions (i.e., are highly exposed to EIMD) present elevated levels of protection against EIMD (Chen et al., 2011; Lima and Denadai, 2011). Thus, it seems reasonable to assume that ISOs would not pose as an intense enough stimuli to promote protection in this case. Figure 1 address this issue, hypothesizing that muscle groups that are highly exposed to EIMD (e.g., trained and/or lower limb muscles) require a greater number of ISOs in order to benefit from a protective effect—with a lesser magnitude and duration—and that EIMD is required for it to manifest. Also, kinetic chain specificity might be an aspect to consider before applying this protection strategy, since open kinetic chain ISOs might not protect against closed kinetic chain-induced damage. Therefore, new investigations should be addressed to better elucidate the underlying aspects behind the protection against EIMD conferred by ISOs.

## Conclusion

When considering applying prophylactic activities, professionals should consider the context of their patients/athletes. For instance, when their primary goal is to avoid strength loss in athletes, ISOs might not be the best preconditioning activity, since it does not seem to be as protective against this EIMD symptom as MaxECC (and not as long lived). For these individuals, performing seasonal MaxECC during off-season or non-competitive moments of the calendar seem to present a more potent and long-lived protection. However, contexts where soreness and other non-strength related EIMD markers are unwanted and the protection does not need to last longer than 4 days (e.g., initiating patients that might be discouraged by swelling and soreness), ISOs seem to be an effective option. Some factors that have been reviewed in the present study should be considered before the adoption of this prevention strategy. It appears that the volume of ISOs play an important role in subsequent protection. However, an elevated number of ISOs might lead to EIMD. It is safe to assume that 10 ISOs are sufficient to promote protection without eliciting EIMD in untrained populations. Additionally, the stimulation-to-damaging bout interval should not exceed 4 days. In fact, a 2-day interval might be ideal. Also, ISOs should be performed at long muscle lengths in order to confer protection. Finally, it is important to consider that protection conferred by ISOs can play a confounding role during the assessment of EIMD markers. In studies that investigate EIMD, strength is usually assessed through 2–5 ISOs. When put together, these contractions might also confer a residual protection when familiarizations and baseline assessments are performed 2–4 days before the damaging bout.

## Conflict of interest statement

The authors declare that the research was conducted in the absence of any commercial or financial relationships that could be construed as a potential conflict of interest.
